# Therapist effects in real-world rehabilitation outcomes: a cohort study of the nationwide GLA:D osteoarthritis management programme in Denmark

**DOI:** 10.1136/rmdopen-2026-007100

**Published:** 2026-06-24

**Authors:** Philips Edomwonyi Obasohan, Joe Palmer, David Alderson, Dahai Yu, Dorte T Grønne, Ewa M Roos, Søren T Skou, George M Peat

**Affiliations:** 1 Department of Liberal Studies, College of Administrative and Business Studies, Niger State Polytechnic, Bida, Nigeria; 2 Centre for Applied Health & Social Care Research (CARe), Sheffield Hallam University, Sheffield, UK; 3 School of Health & Social Care, Sheffield Hallam University, Sheffield, UK; 4 School of Medicine, Keele University, Keele, UK; 5 Center for Muscle and Joint Health, Department of Sports Science and Clinical Biomechanics, University of Southern Denmark, Odense, Denmark; 6 The Research and Implementation Unit PROgrez, Department of Physiotherapy and Occupational Therapy, Central and West Zealand Hospital, Slagelse, Denmark; 7 Department of Research, Central and West Zealand Hospital, Slagelse, Denmark

**Keywords:** Osteoarthritis, Rehabilitation, Health services research, Patient Reported Outcome Measures

## Abstract

**Objective:**

Unlike several other fields of healthcare, little is known about the size of ‘therapist effects’ on patient outcomes following rehabilitation for musculoskeletal conditions. We aimed to estimate the proportion of variance in patient outcomes from a structured rehabilitation programme explained by therapist effects.

**Methods:**

For our observational cohort study, we accessed data from the national multicentre Good Life with osteoArthritis in Denmark (GLA:D) osteoarthritis management programme. Analyses included 23 021 consecutive eligible adults with hip or knee osteoarthritis (mean (SD) age 65.0 (9.8) years, 71% female) treated by 657 therapists between October 2014 and February 2019. The primary outcome was ≥30% reduction in pain intensity on 0–100 visual analogue scale (VAS) at 3 months. Therapist effects were estimated as the variance partition coefficient (intraclass correlation coefficient (ICC)) from two-level random intercept logistic regression models before and after adjusting for patient-level case-mix factors and therapist-level characteristics (number of patients treated, days since therapist certification). Analyses were repeated for a range of secondary outcomes using multiply imputed data and complete-case analysis.

**Results:**

52% of patients reported a ≥30% reduction in pain intensity on 0–100 VAS at 3 months. In the null model, the ICC was 0.007 (95% CI 0.005 to 0.009), which changed little after adjusting for patient-level and therapist-level covariates. Upper confidence limits for ICC estimates across all secondary outcomes in multiply imputed data and complete-case analyses were <0.03.

**Conclusions:**

In a nationally implemented osteoarthritis management programme delivered by trained healthcare professionals, therapist effects made a minimal contribution to variation in patient outcomes.

**WHAT IS ALREADY KNOWN ON THIS TOPIC**

- ‘Therapist effects’—defined as the effect of a given therapist on patient outcomes as compared with another therapist—have been observed in several fields of healthcare and have important consequences for selection, training and service improvement.
- In musculoskeletal rehabilitation, five previous studies suggest that 1%–12% of variation in patient-reported outcomes may be attributable to therapist effects, but these estimates were based on relatively small datasets resulting in substantial uncertainty.

**WHAT THIS STUDY ADDS**

- Our cohort study analysed registry data from 2014 to 2019 on 23 021 patients and 647 trained therapists from the nationally implemented Good Life with osteoArthritis in Denmark structured osteoarthritis management programme in Denmark.
- We found that therapist effects accounted for <3% of total variation in patient-reported pain and quality of life outcomes 3 months after beginning the programme.

**HOW THIS STUDY MIGHT AFFECT RESEARCH, PRACTICE OR POLICY**

- Our findings suggest that contextual factors that relate to therapist effects—therapist characteristics or therapist-patient interaction and alliance—make a minimal contribution to variation in patient outcomes from this structured, group-based rehabilitation intervention.
- Any contextual effects must be attributable to alternative sources, for example, patient expectations, intervention setting.

## Introduction

 The range, difficulty and variability of behaviours required of health professionals are recognised as key mechanisms through which many complex healthcare interventions produce patient benefit.^1^ Therefore, studies that set out to estimate the magnitude of ‘therapist effects’—defined as the effect of a given therapist on patient outcomes as compared with another therapist^2^—have the potential to drive important new insights and lead to improvements in care: Johns *et al*^3^ list four potential ways that studies of therapist effects contribute to knowledge and care: they temper an overemphasis on ‘brands’ of treatment; they identify more or less effective therapists with implications for matching patients, as well as selection, training and revalidation; they contribute to mechanistic understanding and they generate research questions designed to reduce unwarranted variability in service provision and outcomes.

In psychotherapy, surgery and medicine, where there is a relatively long history of such studies, there is strong evidence that therapist effects contribute to patient outcomes, although estimates vary substantially. While extreme estimates suggest that between 0% and 47% of the variation in patient outcomes may be attributable to therapist effects, the more plausible range consistently observed is 3%–10%.^3^,^7^ Factors such as the volume of procedures undertaken by individual surgeons^8^ or psychotherapists’ interpersonal skills^9^ have been proposed as potential determinants.

A comparable body of evidence is largely absent for musculoskeletal rehabilitative interventions. We found only five studies, conducted over the last 15 years, which have estimated therapist effects in this field (table 1).^10^,^14^

**Table 1:** Previously published estimates of therapist effects in musculoskeletal rehabilitation

Study	Study design	Setting	Participants	Therapists/Practitioners	Intervention(s)	Outcomes	Model	ICC/VPC	Comments
Lewis *et al*^10^	Secondary analysis of three RCTs	Physiotherapy services (UK)	350 adults with neck pain	38 PTs	Multiple (advice and exercise; manual therapy; pulsed shortwave diathermy)	Self-reported functional limitation (NPDQ) and psychological health (SF-12 MH) at 6w, 6m	Multilevel (patients nested in therapists) linear and logistic regression models applied to each outcome and end point	0.008–0.071	Greater therapist effect hypothesised when treatment was standardised
		Primary care (NL)	314 adults with low back pain	54 GPs	Psychosocial intervention; usual care	Self-reported functional limitation (RMDQ) and psychological health (FABQ) at 6w, 3m, 12m		0.012–0.08	
		Primary care (UK)	402 adults with low back pain	6 PTs	Brief psychologically informed pain management; manual therapy	Self-reported functional limitation (RMDQ) and psychological health (TSK) at 3m, 12m		0.027–0.051	
Simon *et al*^13^	Observational cohort	Outpatient physical therapy (USA)	258 adults with neck pain or low back pain	5 PTs	Usual care: multiple treatments (mainly manual therapy)	Self-reported pain intensity (VAS) and function (CCFOI) at discharge	Multilevel (patients nested in therapists) linear regression models	≤0.035	No model convergence for pain
Buining *et al*^12^	Observational cohort	Primary care (NL)	393 adults with non-reversible NCD (CVD, RA, orthopaedic, neurological; including 180 with OA)	39 PTs	Usual care	Self-reported symptom severity (0–10 NRS) at end of treatment	Multilevel (patients nested in therapists) linear regression model	0.076	Outcome positively associated with low PT neuroticism
Kooijman *et al*^11^	Observational cohort	Primary care (NL)	1013 adults with rotator cuff pain	46 PTs	Usual care	Change in self-reported severity of complaint (0–10 NRS) between start and end of treatment	Multilevel (patients nested in therapists) linear regression model	0.12	Outcome positively associated with PT extraversion
Nudelman *et al*^14^	Observational cohort	Outpatient physical therapy (ISR)	1043 adults with low back pain	68 PTs	Usual care	Self-reported FS score (0–100 LCAT) and residual (risk-adjusted) FS score at discharge	Multilevel (patients nested in therapists) linear regression model	0.10 (FS score) 0.04 (residual FS score)	Outcome negatively associated with PT biomedically oriented attitude but clinical relevance questionable

CCFOI, CareConnections Functional Outcomes Index; CVD, cardiovascular disease; FABQ, Fear-Avoidance Beliefs Questionnaire; FS, Functional Scale; GP, general practice/practitioner; ICC, intraclass correlation coefficient; ISR, Israel; LCAT, lumbar computerised adaptive test; m, months; NCD, non-communicable disease; NL, The Netherlands; NPDQ, Northwick Park Disability Questionnaire; NRS, Numerical Rating Scale; OA, osteoarthritis; PT, physical therapist/physiotherapist; RA, rheumatoid arthritis; RCT, randomised controlled trial; RMDQ, Roland-Morris Disability Questionnaire; SF-12 MH, 12-item Short Form Health Survey Mental Health component; TSK, Tampa Scale of Kinesiophobia; VAS, visual analogue scale; VPC, variance partition coefficient; w, weeks.

None met the suggested requirement for multilevel models of therapist effects that studies include outcomes from at least 100 healthcare professionals treating at least 10 patients each.^3^ One reason for the apparent paucity of evidence in musculoskeletal rehabilitation has been a lack of available large-scale registry data that combine the collection of standardised patient outcome measures and unique identifiers for therapists. The national rollout of structured osteoarthritis (OA) management programmes with accompanying registry data created the opportunity to examine therapist effects in this field. The nationwide Good Life with osteoArthritis in Denmark (GLA:D) programme is an 8-week structured, group-based, physiotherapist-led OA management programme for people with hip or knee OA comprising two to three patient education sessions and 12 clinician-supervised exercise therapy sessions. Since beginning in 2013, tens of thousands of patients have taken part in programmes delivered by trained physiotherapists across Denmark, mainly in primary care centres and municipal settings.^15^

Given the unique opportunity afforded by the de-identified data from the GLA:D registry, our aim was to estimate ‘real-world’ therapist effects on patient-reported outcomes from a structured, supervised group-based musculoskeletal rehabilitation intervention.

## Methods

### Data source

The Danish national, electronic GLA:D registry houses data on participant characteristics and outcomes collected at baseline, 3 months and 12 months via a combination of patient-reported, therapist-reported and objective measures, and the routine collection of standard outcomes is an integral component of the GLA:D programme.

### Population

For the current analysis, all consecutive participants with hip or knee OA enrolled on the GLA:D programme in Denmark between 9 October 2014 and 28 February 2019 were potentially eligible. These dates represent a period before COVID-19 during which the outcome measures, exposures and covariates of interest in this analysis were included in the data-collection instruments. Participants who had not returned a patient-reported questionnaire at baseline or who did not have a completed therapist ID were excluded from our analyses. We included only participants with a baseline pain intensity score ≥40 out of 100, a common eligibility criterion for clinical trials in OA.^16 17^ For participants taking the programme more than once, only the first (index) attendance was included in the analysis.

### Outcomes

The primary outcome of interest was clinically important pain reduction at 3 months, defined as ≥30% reduction in pain intensity (0–100 visual analogue scale (VAS)) between baseline and 3 months.^17 18^ Secondary outcomes were ≥50% reduction in pain intensity (0–100 VAS), pain intensity score (0–100 VAS) at 3 months, Hip Osteoarthritis Outcome Score (HOOS)/Knee Osteoarthritis Outcome Score (KOOS) quality of life subscale score (0–100) at 3 months, EuroQol 5-Dimension 5-Level (EQ-5D-5L) health utility score (Danish value set, −0.757–1.000) at 3 months and EQ-5D health-related quality of life VAS (0–100) at 3 months. Pain and quality of life (hip/knee-related and general health-related) represent two of the five domains for core outcome sets in hip and knee OA trials (the others being physical function, patient global assessment and adverse events),^19 20^ and are among highly rated optional recommended domains for evaluating OA management programmes.^21^

### Therapists

All certified healthcare practitioners delivering the GLA:D intervention complete 2-day standardised training. The majority are physiotherapists. Analyses were restricted to therapists who had treated at least 10 patients.^3^ Detailed information on therapists is not routinely collected. However, we used cumulative number of patients treated and number of days since completing GLA:D training as indicators of therapist experience.

### Patient-level covariates for case-mix adjustment

Differences in case-mix may explain apparent therapist effects. For case-mix adjustment in our analyses, we identified the following potential patient-level determinants of pain outcome from previous literature^22^ and previous analyses of GLA:D data^23^,^26^: patient age, sex, born in Denmark, Danish citizen, month and year of entry to GLA:D programme, most affected joint (hip/knee), body mass index (BMI) (kg/m^2^), duration of symptoms, walking speed time during 40 m walk test at baseline (m/s), number of chairs stands completed in 30 s at baseline, number of painful body areas at baseline (0–56), University of California, Los Angeles physical activity score at baseline (1–10), EQ-5D VAS at baseline (0–100), EQ-5D-5L health utility score (Danish value set −0.757–1.000), KOOS/HOOS quality of life score at baseline (0–100), Arthritis Self-Efficacy Scale pain and other subscale scores (10–100), SF-12 Physical and Mental Health Component Scores (0–100), pain intensity (0–100) at baseline.

### Statistical analysis

The rate and pattern of missing data were evaluated using the *naniar* package in R. Rates of missing data ranged from 0% to 30% (HOOS/KOOS QOL score at 3 months) due mainly to loss to follow-up (27%) and removal/later inclusion of symptom duration and SF-12 baseline between 2014 and 2019 (ie, missing by design) (online supplemental data). Our primary analysis was of multiply imputed data, judging that complete-case analysis or simple imputation were insufficient due to the risk of bias.^27^ Imputation models were based on the assumption of data missing at random (MAR). Given the concentration of missingness in a few variables, we analysed patterns and probabilities of missing data to identify predictors for missing values.^28 29^ The imputation model included all variables in the final model, including outcome and auxiliary variables (secondary outcomes).^30^ Missing data were imputed using multiple imputation with chained equations using the *mice* package in R to create 40 imputed datasets, to equal or exceed the fraction of missing data^31^ and using 10 iterations each. To achieve model convergence, continuous predictors were standardised and centred.

**Figure 1:**
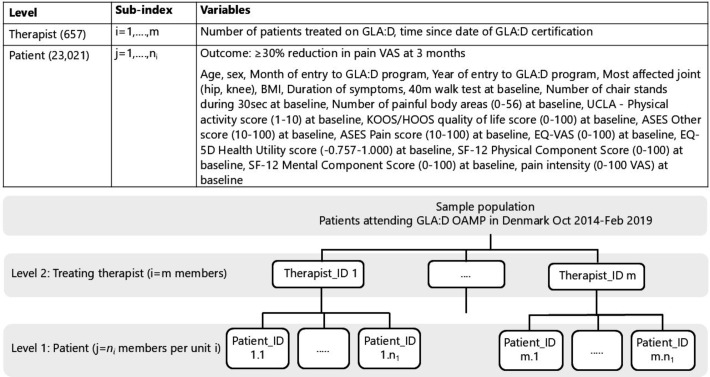
Structure of multilevel model (two levels, primary outcome). ASES, Arthritis Self-Efficacy Scale; BMI, body mass index; EQ-5D, EuroQol 5-Dimension; GLA:D, Good Life with osteoArthritis in Denmark; HOOS, Hip Osteoarthritis Outcome Score; KOOS, Knee Osteoarthritis Outcome Score; OAMP, osteoarthritis management programme; SF-12, 12-item Short Form Health Survey; UCLA, University of California, Los Angeles; VAS, visual analogue scale.

For binary outcomes, we fitted two-level (patients at level 1 nested within therapists at level 2) random intercept logistic regression models (figure 1). Three models were fitted. Model 0 represents the ‘null’ model with no covariates. Model 1 includes the patient-level (level 1) covariates. Model 2 is the full model consisting of model 1 and the therapist-level (level 2) covariates. In each model, we reported the variance partition coefficient estimated by the intraclass correlation coefficient (ICC) using the latent variable method, together with model fit statistics (Akaike Information Criterion (AIC), Bayesian Information Criterion (BIC) and area under the receiver operating characteristic curve (AUC)). For continuous secondary outcomes, random-intercept linear regression models were fitted in the same sequence and using AIC and BIC to evaluate model fit.

To visually display variation between therapists in patient outcomes, we produced funnel plots of the case-mix-adjusted outcomes (therapist-specific observed−expected outcome+overall mean outcome) by number of patients seen per therapist. 95% and 99.8% confidence limits were corrected for an overdispersion factor (phi), calculated using observed variance/expected variance.^32 33^

We compared the above models and outputs from multiply imputed data with the same analyses conducted on a complete-case dataset of therapists with at least 10 patients, each with fully observed data on all predictors and outcomes.

As a final step, we conducted an exploratory descriptive comparison of patient outcomes for selected anonymised therapists who were identified as potential outliers from the above funnel plots.

Analyses were conducted in R V.4.4.0. A list of packages used in R is provided in online supplemental data.

### Patient and public involvement

Patients and members of the public were not involved in the conceptualisation, design, analysis, interpretation or dissemination of this study.

## Results

Table 2 provides the descriptive characteristics of 657 therapists and 23 021 patients included in the current analysis. The median number of patients per therapist was 26 (IQR 16, 44) and therapists were a median of 640 days postcertification (IQR 305, 1073). Patients had a mean (SD) age of 65.0 (9.8) years, 71% were female and they had typically attended higher (postsecondary) education (59%), and most were either retired (53%) or employed/student (29%). The knee joint was the primary complaint in 74% of patients.

**Table 2:** Descriptive characteristics of patients (n=23 021) and therapists (n=657)

	Valid N	Miss %	N	%
Therapists				
Number of patients treated: median (IQR)	657	0	36 (16, 72)	
Days since therapist certification: median (IQR)	657	0	645 (313, 1077)	
Patients				
Age (years): mean (SD)	23 021	0	65.0 (9.8)	
Female sex	23 021	0	17 177	74.4
Educational level	22 994	<1		
Primary			4745	20.6
Secondary			2651	11.5
Higher—short			4705	20.5
Higher—mid			8819	38.4
Higher—long			2073	9.0
Employment status	23 020	<1		
Currently employed/student			6737	29.3
Full-time sick leave			664	2.9
Part-time sick leave			691	3.0
Retired			12 248	53.2
Unemployed			558	2.4
Self-imposed early retirement			1226	5.3
Early retirement due to workability issues			896	3.9
Born in Denmark	23 009	<1	22 081	96.0
Danish citizen	23 009	<1	37 768	98.3
Body mass index (kg/m^2^): mean (SD)	22 956	<1	29.0 (5.5)	
Symptom duration (months): median (IQR)	20 408	11	18 (6, 48)	
40 m walk test at baseline (m/s): mean (SD)	21 792	5	1.4 (0.3)	
Chair test at baseline (no): mean (SD)	22 158	4	11.4 (3.8)	
No of pain areas at baseline: mean (SD)	22 851	<1	4.0 (3.3)	
UCLA activity score (0–10): mean (SD)	23 004	<1	5.5 (1.8)	
Primary complaint	23 019	<1		
Hip			5928	26.0
Knee			17 091	74.0
Month of enrolment on GLA:D	23 021	0		
January			2618	11.4
February			1747	7.6
March			1907	8.3
April			1985	8.6
May			1742	7.6
June			1313	5.7
July			1012	4.4
August			2895	12.6
September			2221	9.6
October			2727	11.8
November			1602	7.0
December			1252	5.4
Year of enrolment on GLA:D	23 021	0		
2014			1214	5.3
2015			3342	14.5
2016			5147	22.4
2017			4981	21.6
2018			4480	19.5
2019			3857	16.8
Type of organisation	23 021	0		
Public clinic			4042	17.6
Private clinic			18 974	82.4
Private hospital			5	<1
Arthritis Self-Efficacy Scale: pain at baseline (10–100): mean (SD)	23 002	<1	61.9 (20.0)	
Arthritis Self-Efficacy Scale: other at baseline (10–100): mean (SD)	22 998	<1	66.6 (18.3)	
SF-12 Physical Component Summary at baseline (0–100): mean (SD)	19 076	17	35.3 (8.2)	
SF-12 Mental Component Summary at baseline (0–100): mean (SD)	19 076	17	51.0 (10.1)	
Pain intensity at baseline (0–100): mean (SD)	23 021	0	61.3 (14.5)	
Pain intensity at 3 months (0–100): mean (SD)	16 746	27	41.4 (22.6)	
Experienced ≥30% reduction in pain at 3 months	16 746	27	8759	52.3
Experienced 50% reduction in pain at 3 months	16 746	27	5750	34.3
HOOS/KOOS QOL at baseline (0–100): mean (SD)	22 215	4	40.9 (14.3)	
HOOS/KOOS QOL at 3 months (0–100): mean (SD)	16 221	30	47.8 (16.6)	
EQ-5D VAS at baseline (0–100): mean (SD)	23 003	<1	64.8 (19.0)	
EQ-5D VAS at 3 months (0–100): mean (SD)	16 748	27	69.7 (19.2)	
EQ-5D utility score at baseline (−0.757–1.000): mean (SD)	22 996	<1	0.710 (0.216)	
EQ-5D utility score at 3 months (−0.757–1.000): mean (SD)	16 743	27	0.780 (0.204)	

Figures represent n (%) unless otherwise stated.

Miss %=rate of missing data for each variable.

EQ-5D, EuroQol 5-Dimension; GLA:D, Good Life with osteoArthritis in Denmark; HOOS, Hip Osteoarthritis Outcome Score; KOOS, Knee Osteoarthritis Outcome Score; QOL, quality of life; SF-12, 12-item Short Form Health Survey; UCLA, University of California, Los Angeles; VAS, visual analogue score.

Pain intensity (0–100 VAS) at baseline was typically moderate to severe (mean 61.3, SD 14.5). Of 16 746 participants with pain intensity scores available at baseline and 3 months, 8759 (53%) had experienced a 30% or greater reduction in pain intensity at 3 months; 5750 (34%) experienced a 50% or greater reduction in pain intensity.

In the VPC model for the primary outcome in multiply imputed data, the estimated ICC was 0.007 (95% CI 0.005 to 0.009) (table 3).

**Table 3:** Random intercept logistic regression models for ≥30% pain reduction at 3 months: multiply imputed data

	Model 0	Model 1	Model 2
	‘Null’ model	Patient-level adjustment model	‘Full model’
	N=23 021	N=23 021	N=23 021
		aOR (95% CI)	aOR (95% CI)
**Therapist-level variables**			
Number of patients treated			0.97 (0.93 to 1.02)
Days since therapist certification			0.99 (0.94 to 1.04)
**Patient-level variables**			
Age		0.94 (0.90 to 0.97)	0.94 (0.90 to 0.97)
Female sex		1.11 (1.03 to 1.19)	1.11 (1.03 to 1.20)
Born in Denmark		1.05 (0.85 to 1.29)	1.04 (0.85 to 1.29)
Danish citizen		1.05 (0.76 to 1.46)	1.05 (0.76 to 1.46)
Month of enrolment on GLA:D (ref: January)			
February		0.96 (0.83 to 1.11)	0.96 (0.83 to 1.11)
March		1.12 (0.98 to 1.29)	1.13 (0.98 to 1.30)
April		1.08 (0.95 to 1.24)	1.08 (0.95 to 1.24)
May		1.16 (1.00 to 1.34)	1.16 (1.01 to 1.35)
June		1.07 (0.91 to 1.26)	1.08 (0.92 to 1.27)
July		1.05 (0.89 to 1.24)	1.06 (0.89 to 1.25)
August		1.09 (0.95 to 1.24)	1.09 (0.96 to 1.25)
September		1.07 (0.93 to 1.22)	1.07 (0.94 to 1.23)
October		1.13 (0.99 to 1.28)	1.14 (1.00 to 1.29)
November		1.15 (0.99 to 1.33)	1.16 (1.00 to 1.34)
December		0.94 (0.80 to 1.10)	0.95 (0.81 to 1.12)
Year of enrolment on GLA:D (ref: 2014)			
2015		0.94 (0.81 to 1.10)	0.95 (0.82 to 1.11)
2016		0.88 (0.76 to 1.02)	0.90 (0.78 to 1.05)
2017		1.00 (0.86 to 1.16)	1.04 (0.89 to 1.21)
2018		1.05 (0.90 to 1.23)	1.11 (0.94 to 1.31)
2019		0.97 (0.83 to 1.14)	1.03 (0.86 to 1.24)
Most affected joint is hip (ref: knee)		0.82 (0.77 to 0.88)	0.82 (0.77 to 0.88)
BMI (kg/m^2^)		0.95 (0.92 to 0.99)	0.95 (0.92 to 0.99)
Duration of symptoms (months)		0.91 (0.88 to 0.94)	0.91 (0.88 to 0.94)
40 m walk test at baseline		1.10 (1.05 to 1.14)	1.10 (1.05 to 1.15)
No of chair stands during 30 s at baseline		1.05 (1.01 to 1.09)	1.05 (1.01 to 1.09)
No of painful body areas (0–56) at baseline		0.92 (0.88 to 0.95)	0.92 (0.88 to 0.95)
UCLA—physical activity (1–10) at baseline		0.99 (0.96 to 1.02)	0.99 (0.96 to 1.02)
KOOS/HOOS QOL (0–100) at baseline		1.11 (1.06 to 1.15)	1.11 (1.06 to 1.15)
ASES: other (10–100) at baseline		0.97 (0.92 to 1.03)	0.97 (0.92 to 1.03)
ASES: pain (10–100) at baseline		1.26 (1.20 to 1.33)	1.26 (1.20 to 1.33)
EQ-VAS (0–100) at baseline		1.08 (1.04 to 1.13)	1.08 (1.04 to 1.13)
EQ-5D health utility score (−0.757–1.000) at baseline		1.20 (1.15 to 1.26)	1.20 (1.15 to 1.26)
SF-12 PCS (0–100) at baseline		1.00 (0.96 to 1.05)	1.00 (0.96 to 1.05)
SF-12 MCS (0–100) at baseline		0.94 (0.90 to 0.99)	0.94 (0.90 to 0.99)
Pain intensity (0–100) at baseline		1.63 (1.57 to 1.69)	1.63 (1.57 to 1.69)
VPC (ICC)	0.007 (0.005 to 0.009)	0.008 (0.005 to 0.011)	0.008 (0.005 to 0.011)
AIC	30 263.79	30 263.40	30 263.79
BIC	30 561.43	30 577.13	30 561.43
AUC	0.660	0.661	0.660

All predictors were standardised and centred.

AIC, Akaike Information Criterion; aOR, adjusted OR; ASES, Arthritis Self-Efficacy Scale; AUC, area under the curve; BIC, Bayesian Information Criterion; BMI, body mass index; EQ-5D, EuroQol 5-Dimension; GLA:D, Good Life with osteoArthritis in Denmark; HOOS, Hip Osteoarthritis Outcome Score; ICC, intraclass correlation coefficient; KOOS, Knee Osteoarthritis Outcome Score; MCS, Mental Component Summary; PCS, Physical Component Summary; QOL, quality of life; ref, reference; SF-12, 12-item Short Form Health Survey; UCLA, University of California, Los Angeles; VAS, visual analogue score; VPC, variance partition coefficient.

Baseline patient characteristics associated (p<0.05) with increased odds of achieving a ≥30% reduction in pain at 3 months were: younger age, female sex, primary complaint was knee problem, lower BMI, shorter duration of complaint, faster walking speed, greater number of chair stands, fewer areas of body pain, higher arthritis self-efficacy scores, higher HOOS/KOOS quality of life subscale scores, higher EQ-5D VAS scores, lower SF-12 Physical Component Summary scores, higher SF-12 Mental Component Summary scores and higher pain intensity. Although the AIC remained constant across the three models, the BIC for model 2 was lower than that for model 1, indicating that the addition of therapist-level characteristics did improve model fit. However, therapist-level predictors—cumulative number of patients treated (aOR 0.97 (95% CI 0.93 to 1.02)) and number of days since completing GLA:D training (0.99 (0.94 to 1.04))—were not statistically significantly associated with the outcome. The ICC (0.007) in the null model was statistically significant and effectively unchanged after adjusting for patient-level characteristics (0.008) and therapist-level characteristics (0.008). The AUC of 0.66 suggests weak discriminative ability.

**Figure 2:**
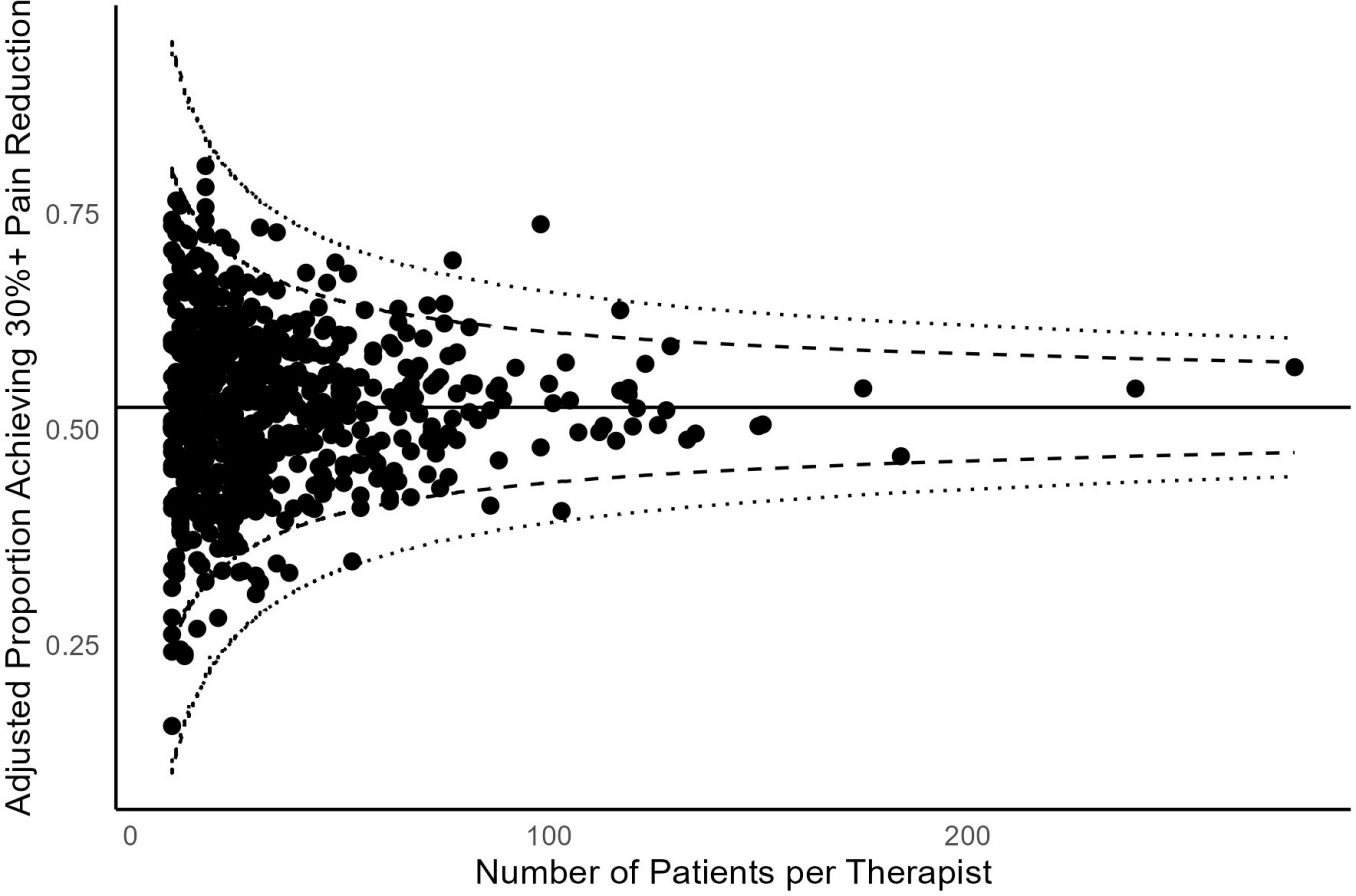
Funnel plot of case-mix adjusted pain relief outcomes by therapist: Good Life with osteoArthritis in Denmark 2014–2019.

The funnel plot of therapist-specific outcomes, adjusted for patient-level and therapist-level covariates and corrected for overdispersion, identified two outlier therapists with better-than-expected outcomes at the 99.8% confidence threshold (figure 2).

Across all secondary outcomes and models in the multiply imputed data analyses, the pattern of predictor-outcome associations and the magnitude of the ICC was similar (table 4, online supplemental data).

**Table 4:** Summary of ‘therapist effects’ for primary and secondary outcomes, multiply imputed data and complete-case analysis

	‘Null’ model	Patient-level adjustment model	‘Full model’
	N=23 021	N=23 021	N=23 021
	ICC (95% CI)	ICC (95% CI)	ICC (95% CI)
Outcome: proportion of patients achieving 30% reduction in pain intensity at 3 months			
Multiply imputed data (n=23 021)	0.007 (0.005 to 0.009)	0.008 (0.005 to 0.011)	0.008 (0.005 to 0.011)
Complete-case analysis (n=9720)	0.014 (0.005 to 0.026)	0.015 (0.005 to 0.025)	0.015 (0.005 to 0.024)
Outcome: proportion of patients achieving 50% reduction in pain intensity at 3 months			
Multiply imputed data (n=23 021)	0.007 (0.004 to 0.009)	0.007 (0.004 to 0.010)	0.007 (0.004 to 0.010)
Complete-case analysis (n=9720)	0.012 (0.002 to 0.022)	0.013 (0.003 to 0.024)	0.013 (0.001 to 0.022)
Outcome: pain intensity score (0–100) at 3 months			
Multiply imputed data (n=23 021)	0.008 (0.006 to 0.010)	0.009 (0.006 to 0.011)	0.009 (0.006 to 0.011)
Complete-case analysis (n=9720)	0.015 (0.006 to 0.023)	0.016 (0.005 to 0.025)	0.016 (0.007 to 0.022)
Outcome: HOOS/KOOS quality of life subscale score (0–100) at 3 months			
Multiply imputed data (n=23 021)	0.008 (0.007 to 0.010)	0.006 (0.005 to 0.008)	0.006 (0.005 to 0.008)
Complete-case analysis (n=9720)	0.014 (0.006 to 0.026)	0.012 (0.002 to 0.022)	0.012 (0.003 to 0.022)
Outcome: EQ-5D health utility score (−0.757–1.000) at 3 months			
Multiply imputed data (n=23 021)	0.008 (0.006 to 0.010)	0.003 (0.001 to 0.005)	0.003 (0.001 to 0.005)
Complete-case analysis (n=9720)	0.012 (0.001 to 0.022)	0.008 (0.001 to 0.016)	0.008 (0.000 to 0.019)
Outcome: EQ-5D VAS score (0–100) at 3 months			
Multiply imputed data (n=23 021)	0.011 (0.009 to 0.013)	0.004 (0.002 to 0.006)	0.004 (0.002 to 0.005)
Complete-case analysis (n=9720)	0.015 (0.001 to 0.026)	0.008 (0.001 to 0.015)	0.008 (0.002 to 0.018)

EQ-5D, EuroQol 5-Dimension; HOOS, Hip Osteoarthritis Outcome Score; ICC, intraclass correlation coefficient; KOOS, Knee Osteoarthritis Outcome Score; VAS, visual analogue scale.

Complete-case analysis included 9720 participants and 403 therapists with complete data on all outcomes and covariates. Across all outcomes and models, the ICC estimates were systematically higher than in the analyses of multiply imputed data. However, the upper 95% confidence limits for all ICC estimates were below 0.03 (online supplemental data).

## Discussion

### Summary of key findings

The present study used data available from a large-scale national registry to estimate the magnitude of ‘therapist effects’ on short-term patient-reported pain and quality of life outcomes for adults enrolled on a structured, group-based, physiotherapist-led OA management programme. Our multilevel models suggested small or very small therapist effects, accounting for < 3% of the variance across all outcomes, analyses and models.

### Comparison with previous studies

Our estimates are at the lowest end of the range of 1%–12% reported in previous studies of therapist effects in musculoskeletal pain conditions,^10^,^14^ and the 3%–12% range typically observed in other conditions and settings.^3^,^7^ Our estimates are, however, in line with the only previous published ICC estimate of therapist-level clustering of patient outcomes in OA cluster randomised trials.^34 35^ Before speculating on substantive reasons why this might be, it is important to consider whether this could reflect model misspecification and unreasonable assumptions during modelling.^36^

Excluding outlier therapists would be expected to reduce therapist effects. Selection of therapists for inclusion in our analyses was based only on having seen a minimum of 10 patients—a suggested minimum for such analyses.^3^ We were careful not to select or exclude any therapists based on their characteristics or outcomes, including the number of their patients providing incomplete data and missing outcome data at 3 months. Overall, 27% of eligible participants did not have the primary outcome observed, which was expected to vary by therapist, and could not be assumed to be missing completely at random. In these circumstances, complete-case analysis loses statistical power and is likely to be susceptible to bias.^37^ Our primary analysis was therefore based on multilevel multiply imputed data, including imputing missing outcome data. Our findings assume data MAR and correct specification of the multiple imputation procedures. We did not find substantially larger estimates from complete-case analysis, suggesting that any misspecification of the imputation procedures may be unlikely to mask substantially higher ICC values. Nevertheless, it is possible that the (self-)selection and training of therapists to deliver a standardised intervention effectively reduces the amount of therapist variance that is present in the data. Indeed, this would be an intentional goal to ensure the GLA:D programme is delivered in a consistent manner. In studies of psychotherapy, manualised treatment has been associated with lower therapist effects.^38^ Previous studies in musculoskeletal rehabilitation using routine or registry data were largely based on unselected physiotherapists providing an unspecified mixture of treatment approaches under ‘usual care’.^11^,^14^ Lewis *et al*^10^ provide direct comparisons of ICC values between trial treatment arms, including usual care, but the highly variable ICC estimates likely also reflected limited sample sizes for their stratified analyses.

Inappropriate model assumptions and analytic processes may also inflate patient variation, and consequently reduce the contribution of therapist effects to total variation in outcomes or reduce the ability to identify true outliers.

The choice of outcome measure and timing of end point seem unlikely to explain the difference: previous studies have used a mixture of pain/symptom and functional outcomes and end points with no obvious relationship to ICC estimates emerging. ICC estimates from two trials using self-reported functional outcome at 3 months were 2.6%^39^ and 2.7%,^40^ respectively. Our study chose a combination of binary and continuous outcomes across three domains, pain, hip/knee-specific quality of life and health-related quality of life. The magnitude of ICC estimates did not differ substantially from one outcome to another.

### Strengths and limitations

This is the largest study to date of therapist effects in musculoskeletal rehabilitation, made possible by the routine collection and recording of consistent data on patient-reported outcomes and therapists at scale in the nationwide GLA:D programme. It comfortably exceeds the suggested guidance of a minimum of 100 therapists each treating at least 10 patients. We were able to incorporate a large number of patient-level covariates in the case-mix adjustment. Higher pain severity, longer symptom duration, multiple site pain, older age and higher BMI were consistently associated with worse outcomes in the current study. These are well-recognised prognostic indicators of poor outcome in musculoskeletal pain conditions,^41^ although residual confounding from unmeasured patient-level covariates is still possible.

There were some limitations in the data available. ‘Ability to participate in daily activities’ is a core outcome domain for OA management programmes^21^ but KOOS-12 Function was introduced only partway through the period covered in the current analysis. Few therapist-level factors were available within the GLA:D registry data, and this meant it was not possible to extend previous work exploring the importance of personality traits and attitudes for therapist effects^11^,^14^ or to explore other potential mechanisms underlying therapist effects such as treatment fidelity, communication style or wider clinical experience. However, previous empirical analyses of over 1000 outcomes from primary care cluster randomised controlled trials found that adjustment for covariates tended, on average, to reduce ICC estimates.^42^ We therefore suspect that the addition of further therapist-level covariates in our models would not produce substantially larger ICC estimates. Outcome measures were available at 12 months; however, we reasoned that therapist effects would most likely be observed in the short term. An alternative hypothesis might be that some therapists are more effective at engaging patients and creating sustained outcomes. Future studies of long-term outcomes could investigate this. Similarly, it could be of interest to extend our analyses to other outcomes such as functional performance tests. Our choice of 30% reduction in pain intensity for primary analysis is open to challenge. Outcome Measures in Rheumatology-Osteoarthritis Research Society International (OMERACT-OARSI) criteria,^43^ for example, consider 20% improvement (coupled with improvement in function or patient global assessment) as clinically important improvement. However, our secondary analyses included a higher threshold (50%) and pain intensity as a continuous outcome, and found consistent ICC estimates. Based on this, we would not expect a change to 20% threshold to alter our findings on therapist effects.

GLA:D is delivered as a group intervention, whereas the previous estimates of therapist effects in musculoskeletal rehabilitation have come from individual treatment. If some of the variation in patient outcomes is due to interactions between group members that are independent of the therapist, this may have contributed to the lower estimates of therapist effects observed in the current study.

### Implications for research and/or practice

Implications of our findings depend to some extent on whether therapist effects are viewed as contextual or specific effects, and whether we believe that selection and training of therapists for GLA:D, together with standardisation of the GLA:D intervention, have effectively minimised the scope for variation in therapist effects on patient outcomes. Therapist effects may be viewed as part of wider concepts of contextual effects, often additive to ‘specific’ treatment effects.^44^ Substantial contextual factors are seen across most treatments for OA,^45 46^ and seem likely also for exercise-based interventions.^47^ Our findings extend this work by suggesting that contextual factors that relate to therapist effects—therapist characteristics or therapist-patient interaction and alliance^48^—make a minimal contribution to variation in patient outcomes from this structured, group-based rehabilitation intervention. Any contextual effects must be attributable to alternative sources, for example, patient expectations, intervention setting.

Therapist effects may also be viewed as integral to ‘specific’ treatment effects.^49 50^ The recently developed core capability framework for qualified healthcare professionals asserts that health professionals require a diverse array of skills to provide optimal care, including rehabilitative interventions, for all people with OA.^51^

The lack of therapist effects seen in our study could be consistent with two alternative explanations: either these capabilities are fairly consistently present among all therapists delivering the GLA:D intervention or that patient outcomes from this structured programme are not particularly dependent on them. Determining between these competing explanations has important implications for the selection and training of therapists to deliver structured rehabilitation programmes, perhaps even the expansion of this role to other personnel. The emergence of OA management programmes internationally, with varying structure, context and content, may present opportunities not just for replication of our findings but crucial insights that discriminate between these competing alternative explanations.^52^ At the present time, our study suggests that further efforts to select and match therapists to deliver OA management programmes, or to monitor therapist-level outcomes over and above current practice in GLA:D appear unwarranted.

## Conclusion

National GLA:D registry data provide a unique opportunity to investigate the magnitude of therapist effects on patient outcomes from a structured, group-based OA management programme delivered by trained healthcare professionals. While therapists play important roles in the delivery of OA care, in this context we found that therapist effects—whether viewed as contextual factors or integral to specific treatment effects—made a minimal contribution to variation in patient outcomes.

## Supplementary material

10.1136/rmdopen-2026-007100 online supplemental file 1

## Data Availability

Data may be obtained from a third party and are not publicly available.
